# Genomic Selection Improves Heat Tolerance in Dairy Cattle

**DOI:** 10.1038/srep34114

**Published:** 2016-09-29

**Authors:** J. B. Garner, M. L. Douglas, S. R. O Williams, W. J. Wales, L. C. Marett, T. T. T. Nguyen, C. M. Reich, B. J. Hayes

**Affiliations:** 1 Agriculture Victoria, Department of Economic Development, Jobs, Transport and Resources, 1301 Hazeldean Road, Ellinbank, Victoria 3821, Australia; 2 BioSciences Research, Department of Economic Development, Jobs, Transport and Resources, AgriBio, 5 Ring Road, Bundoora, Victoria 3083, Australia; 3 Queensland Alliance for Agriculture and Food Innovation, Centre for Animal Science, University of Queensland, Queensland, Australia

## Abstract

Dairy products are a key source of valuable proteins and fats for many millions of people worldwide. Dairy cattle are highly susceptible to heat-stress induced decline in milk production, and as the frequency and duration of heat-stress events increases, the long term security of nutrition from dairy products is threatened. Identification of dairy cattle more tolerant of heat stress conditions would be an important progression towards breeding better adapted dairy herds to future climates. Breeding for heat tolerance could be accelerated with genomic selection, using genome wide DNA markers that predict tolerance to heat stress. Here we demonstrate the value of genomic predictions for heat tolerance in cohorts of Holstein cows predicted to be heat tolerant and heat susceptible using controlled-climate chambers simulating a moderate heatwave event. Not only was the heat challenge stimulated decline in milk production less in cows genomically predicted to be heat-tolerant, physiological indicators such as rectal and intra-vaginal temperatures had reduced increases over the 4 day heat challenge. This demonstrates that genomic selection for heat tolerance in dairy cattle is a step towards securing a valuable source of nutrition and improving animal welfare facing a future with predicted increases in heat stress events.

Projected future increases in temperature and humidity could have significant negative implications on the productivity and welfare of ruminants that provide milk, meat and power for people worldwide. In dairy cattle, temperature and humidity (often combined as a temperature humidity index, THI) exceeding species thresholds lead to significant declines in feed intake, milk production, and fertility, which are associated with physiological changes including increased core body temperature, respiration and panting rates, sweating, and endocrine system changes which are important to health and productivity^1^,^2^,^3^. High producing lactating dairy cattle are the most susceptible of domesticated production animals to the deleterious effects of heat stress. A dairy cow’s thermoneutral zone (for *Bos taurus* breeds) is between 0.5 °C to 20 °C^4^. The upper critical ambient temperature for high producing *Bos taurus* dairy cattle at which body temperature begins to rise is approximately 25 °C^5^, though this will vary depending on production status (lactating vs. non-lactating, growing), degree of acclimatisation, pregnancy status, diet and climatic variables including solar radiation, wind speed and relative humidity^6^,^7^.

The frequency and duration of heat-stress conditions in dairying regions is increasing. As an example, analysis of climatic data from temperate dairying regions in southern Australia demonstrates that the total number of heat stress days (THI >75 to 82) has increased for the period from 1960 to 2008. The average number of consecutive days of heat-stress conditions in this region has also increased from 2 days per heat stress event in the period of 1960 to 1999, to 4 days per heat stress event in the period of 2000 to 2008, and this is predicted to increase further into the future^8^. Consecutive days of heat-stress conditions have the most impact, as the opportunity for cattle to dissipate body heat at night is reduced. As a result, the increase in body temperature is accumulated from day to day for the duration of the heat event, leading to significant declines in production and resulting in negative animal health and welfare outcomes^9^. The increased incidence and duration of heat stress events is already impacting milk production in Australia. Amelioration strategies to mitigate the negative effects of heat stress include the provision of shade, sprinklers, fans at the dairy, adequate water and changes to feeding and mustering management to avoid the hottest parts of the day^10^. However, these strategies may not be effective in pasture-based dairy systems as the cows spend the majority of their time exposed to solar radiation while grazing.

The extent of lost production in response to heat stress varies among dairy cows and a proportion of this variation is heritable^11^,^12^. The threshold of environmental conditions that cause declines in production are largely attributed to genetic adaptations and as the climatic conditions exceed the thermoneutral threshold of cow comfort, the genetic variance for heat tolerance increases, which creates scope for selection to improve heat tolerance and production^11^.

Investigating heat stress responses between different dairy breeds is well established^13^,^14^,^15^. In this study we focus on exploiting the variation within breed, which is more relevant for many dairy industries worldwide. Dairy herds are increasingly dominated by just a few breeds, in particular the Holstein breed used in our experiment^16^.

Traditional selective breeding in dairy cattle has slow rates of genetic gain as a result of long generation intervals. Given the rate of increase in days of heat stress, faster genetic gains are desirable. Genomic selection uses genome wide DNA markers to capture the effects of the many mutations that influence variation in a complex trait like heat tolerance, and allows young bulls and heifers to be selected on their genomic estimated breeding values (GEBV), thereby accelerating genetic gain. Genomic predictions for heat tolerance in dairy cattle were developed by combining 11 years of weather station data, herd test day milk yields for more than 366,000 cows, and genomic data (632,003 single nucleotide polymorphism (SNP) markers)^17^. For each cow in their data set, the rate of decline in milk production with increasing THI was estimated. This heat tolerance phenotype was then used to derive genomic predictions for the trait. In this study, we go a step further, and attempt to validate these predictions in a cohort of 24 cows predicted to be heat tolerant (HT) and 24 cows predicted to be heat susceptible (HS) following exposure to heat-stress conditions in controlled-climate chambers.

We hypothesised 1) that by day 4 of the heat challenge, there would be a reduction in milk yield in all cows, but this reduction would be smaller in the predicted HT cows compared to the HS cows, and 2) that the HT cows would have a lesser response to the heat challenge than the HS cows as measured by physiological stress indicators such as core temperature and respiration rate.

To select cows for the experiment, 390 Holstein-Friesian heifers from herds across the main dairying regions of Victoria, Australia were genotyped using the BovineLD BeadChip (Illumina, San Diego) that contains 6909 SNPs, and their SNP data were then imputed to the 632, 003 SNPs found on the Bovine HD array^18^,^19^. The GEBV for heat tolerance were calculated using the prediction equation of Nguyen *et al.*^17^. Cows were ranked on GEBV for heat tolerance, and the 24 predicted most HT and 24 predicted most HS were relocated to the research centre.

The 48 cows were randomly assigned to controlled-climate chambers for a 4 day heat challenge followed by a 14 day recovery period. Cows were also monitored during a 7 day baseline period prior to the heat challenge. Temperature and relative humidity inside the controlled-climate chambers was varied to mimic the diurnal patterns in heat load imposed on dairy cows that occur during heat-wave events in southern Australia. The temperature ranged from 23.3 to 31.6 °C (26.3 °C mean) and relative humidity from 42.2 to 71.2% RH (55.2% mean), (THI = 71.6 to 82.1, 75.4 mean). The 12 h light (0600 to 1800 h) and dark cycle was controlled manually (Supplementary Figure 1). During the baseline and recovery periods the cows experienced ambient conditions where temperature ranged from 3 to 23.4 °C (mean 10.4 °C) and relative humidity ranged from 43.7 to 98.1% (mean 82.5%).

Preceding the experiment, thresholds of severe heat stress were set for respiration rate, panting score and rectal temperature (see methods for thresholds). If these parameters were exceeded the affected cow would be removed from the heat challenge. During the experiment three HS cows were removed from the heat challenge and no HT cows exceeded the thresholds for these parameters.

## Results and Discussion

**Figure 1:**
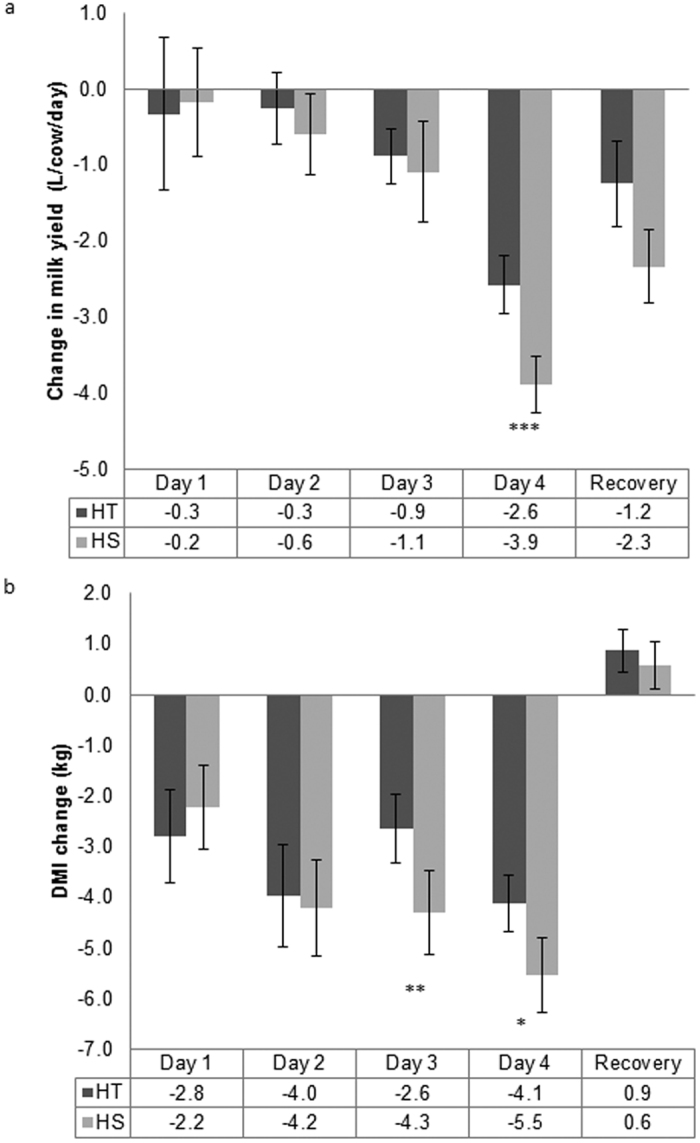
Changes from baseline in mean daily milk yield and feed intake of the HT and HS cows over a four day heat challenge and 14 day recovery period with standard error bars. (**a**) Milk yield change from baseline. (**b**) Dry matter intake (DMI) change from baseline. Trends identified at ^^^P < 0.10, and statistical difference identified at *P < 0.05, **P < 0.01, and ***P < 0.001.

The amount of milk yield decline was less for the HT cows during the heat challenge than for the HS cows. The decline in daily milk yield from the baseline period to day 4 of the heat challenge was less (P = 0.023) for the HT cows (12.5% decline from baseline) than for the HS cows (17.4% decline from baseline) (Fig. 1a, Supplementary Table 1). The measured decline in milk yield was not different (P = 0.06) to the genomic predicted milk yield decline for both the HT and HS cows. Milk yield, and energy corrected milk (ECM), which is the milk yield calculated to have the same energy content, declined similarly to non-corrected milk yield. From baseline to day 4 of the heat challenge, ECM of the HT cows declined by 8.1% and the ECM of HS cows declined by 15.7%. The effect of baseline milk yield was minimised by balancing the HT and HS groups for milk yield and ECM, and, the baseline milk yield of each group were fitted in the statistical model (see methods) to ensure the test for difference in decline in production was independent of initial milk yield. The differences in the change in milk production traits between the HT and HS cows suggests fundamental differences in energy metabolism between the HT and HS cows. Heat stressed animals are known to have increased energy expenditure for heat loss mechanisms via panting and sweating^7^. It is estimated that during heat stress, the maintenance energy requirements of lactating dairy cows increase by up to 25%^21^. Pair feeding experiments demonstrate that only a portion (35 to 50%) of this heat-stress induced milk yield decline can be explained by reduced nutrient intake^1^,^20^. The remaining portion may be a consequence of energy-intake independent changes in nutrient partitioning and the energy demanding process of thermoregulation.

**Figure 2:**
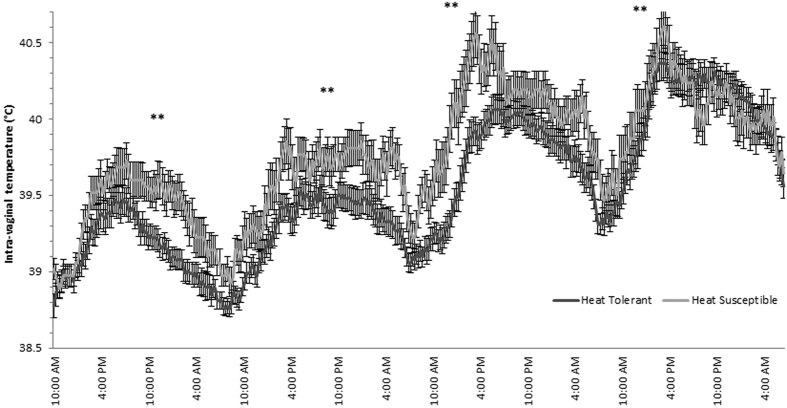
Intravaginal temperature for the HT and HS cows over the four day heat challenge with standard error bars.

**Figure 3:**
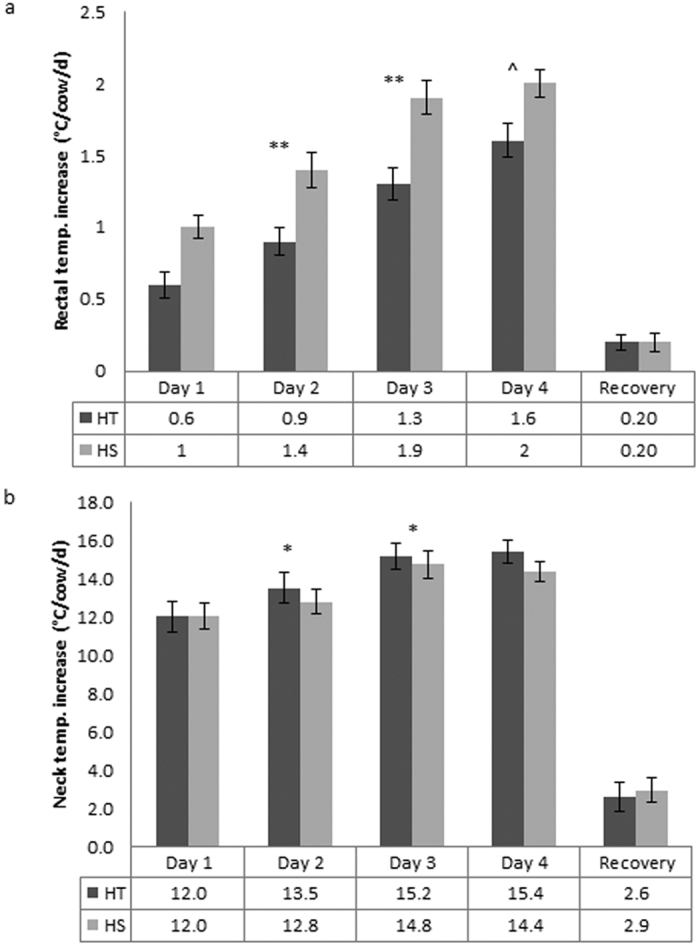
Difference in mean daily physiological parameters for HT and HS cows over a four day heat challenge and 14 day recovery period with standard error bars. (**a)** Rectal temperature increase from baseline, (**b)** Neck temperature increase from baseline.

Core body temperature was lower in HT cows than HS cows throughout the heat challenge, in agreement with our hypothesis. The HT cows had a lower (day 1 P = 0.007, day 2 P = 0.004, day 3 P = 0.002, and day 4 P = 0.007) daily mean vaginal temperature for each day of the heat challenge period than the HS cows (Fig. 2). An area under the curve analysis (AUC) demonstrated that the HT cows maintained their vaginal temperature °C.minutes lower during each day of the heat challenge (day 1 P = 0.057, day 2 P = 0.012, day 3 P = 0.012 and day 4 P = 0.11, Supplementary Table 2). This is supported by the lower mean AM and PM rectal temperature of the HT cows on days 2 (P = 0.004), 3 (P = 0.003) and 4 (P = 0.07) of the heat challenge period than the HS cows (Fig. 3a). The increases in rectal and vaginal temperature recorded in this experiment were consistent with moderate heat stress^22^. This shows that the HT cows consistently displayed superior thermoregulatory ability during the heat challenge by maintaining core body temperatures lower than the HS cows. The observed increases in core body temperature during the heat challenge period indicate that the THI imposed in this experiment was sufficient to cause heat stress in the cows. We chose to mimic typical summer conditions in southern Australia and, although the temperature dropped to 25 °C for 12 hours overnight, the cows still exhibited elevated core body temperature and respiration rates on the morning of each treatment day which indicated that the heat stress was being maintained for the entire 24 hours of each day.

Skin temperature differences were observed between the HT and HS cows. The HT cows had a higher mean AM flank temperatures for days 2 (P = 0.02), 3 (P = 0.015), and 4 (P = 0.05), greater PM mean flank temperatures on days 2 (P = 0.016), 3 (P = 0.07) and 4 (P = 0.01) of the heat challenge, greater daily mean neck temperatures on days 2 (P = 0.039), 3 (P = 0.033), and 4 (P = 0.001), greater AM mean neck temperature on day 4 (P = 0.001), and greater PM neck temperatures on days 2 (P = 0.013), 3 (P = 0.035), and 4 (P = 0.007) (Fig. 3b, Supplementary Table 2), than the HS cows. The differences in skin temperature indicates the HT cows were better at dissipating heat than the HS cows. Heat loss occurs when cooler air comes into contact with a warmer skin surface and draws the heat away from the body via convection currents^23^. Vasodilation is a physical response to heat stress that takes advantage of this heat dissipation method as it draws more heat to the skin surface by increasing blood flow to the periphery^24^. The higher the skin surface temperature the greater the opportunity to utilise the thermal gradient to offload heat to the air. The higher neck and flank temperature of the HT cows compared to that of the HS cows is consistent with superior thermoregulatory ability and is indicative of greater vasodilation by the HT cows. This heat dissipation mechanism may account for the lower rectal and vaginal temperature of the HT cows than the HS cows on each day of the heat challenge.

Respiration rate was lower in the HT cows on day 2 (P = 0.06) and 3 (P = 0.05) of the heat challenge than the HS cows. Given the lower core body temperatures measured in the HT cows, the lower respiration rate may be indicative of the cows expelling more heat via the respiratory tract as air moves over the upper respiratory surface, transferring heat from the blood stream to the air which is then respired. Indeed, for every 1 ml of water evaporated from the respiratory tract surface, 2.43 J of heat is lost which cools the blood flowing through the underlying regions of the respiratory tract^23^. There was, also, a lower panting score observed in the HT cows on days 2 (P = 0.013) and 4 (P = 0.023) of the heat challenge than the HS cows. We speculate that as there is evidence of greater vasodilation and skin surface temperatures of the HT cows, there may have been a greater temperature gradient from the respiratory tract surface of the HT cows which may have led to more heat being dissipated per breath than the HS cows, thus creating more efficient heat dissipation via evaporative cooling and the ability to consistently maintain a lower core body temperature during the heat challenge.

Feed intake declined for all cows during the heat challenge, but the decline was less for the HT cows than the HS cows. The decline in mean daily dry matter intake (DMI) from the baseline period to day 3 (P = 0.04) and 4 (P = 0.08) of the heat challenge was less for the HT cows than for the HS cows (Fig. 1b). Such changes in feed intake are a well-established response to heat stress^1^,^20^. A reduction in DMI is a behavioural adaptive response that reduces metabolic heat production by lowering the heat of fermentation produced by the rumen, and this lowers core body temperature^25^,^26^. Despite the HT consuming more feed than the HS cows, the HT cows had lower core body temperatures during the heat challenge period than the HS cows. This suggests that the differences in core body temperature may not only be influenced by metabolic heat produced by fermentation in the rumen and could be attributed to inherent differences in thermal physiology.

In the recovery period after the heat challenge, feed intake and milk production had not returned to baseline measurements. Feed intake during the recovery period was greater than during the baseline period for both groups. With reference to their baseline DMI, the mean daily DMI during the recovery period was greater by 4.3% for the HT cows and 3.5% for the HS cows (Fig. 1b, Supplementary Table 1). The HT cows returned to their baseline DMI by day 6 of the recovery period while the HS cows returned by day 9. This increase in feed intake was not associated with a return to normal milk yield for either group. Milk yield continued to decline in all cows during the recovery period. Milk production declined from baseline by 5.1% for the HT cows and 10.2% for the HS cows during the recovery period (Fig. 1a, Supplementary Table 1). A similar decline was apparent for ECM, from baseline the decline was 6.1% for the HT cows and 10.9% for the HS cows. We speculate that following the heat challenge the cows were experiencing a period of metabolic recovery as the return to baseline DMI did not see a return to baseline milk production, and based on the differences in the post heat challenge milk yield, the HT cows appeared to be less affected by the heat challenge than the HS cows.

The intake of water by the HT cows was less than that by the HS cows on days 2 (P = 0.02) and 4 (P = 0.026) of the challenge period (Supplementary Table 1). This is important as increases in water consumption during heat stress are positively associated with milk volume^27^, and, with cooling of the body by lowering rumen temperature^28^. The HT cows in this experiment were not consuming more water and therefore the lower core body temperatures and milk yield decline in these cows during the heat challenge cannot be attributed to differences in water consumption.

## Conclusion

In conclusion, we have demonstrated that dairy cattle predicted by genomic breeding values to be heat tolerant, have less decline in milk production, and reduced increases in core body temperature, during a simulated heat wave event in comparison to cows predicted to be heat susceptible. Thus, genomic selection for heat tolerance could increase the resilience and welfare of dairy herds worldwide and the productivity of dairy farming in a future with increased incidence and duration of heat stress events.

## Materials and Methods

### Cows and design

The experiment received animal ethics approval from the Agricultural Research and Extension Animal Ethics Committee of the Department of Economic Development, Jobs, Transport and Resources, Victoria, Australia. All methods were performed by approved staff members in accordance with the relevant standard operating procedures approved by the above mentioned ethics committee. A set of defined thresholds for severe heat stress was defined, and if any cow reached all of the thresholds she was to be removed from the heat challenge. The thresholds were rectal or intra-vaginal temperature of >40.9 °C, panting score of >3 (out of 4.5), and a respiration rate of >100 breaths per minute. The experiment was conducted during winter to early spring (July to October, 2015).

Four hundred primiparious Holstein-Friesian cows were genotyped with the BovineLD BeadChip (Illumina, San Diego, California). Genotypes were imputed to 632,003 high density genotypes using Beagle 3.0^18^,^19^. A small number of animals (10) were excluded from further analysis to avoid breed composition as a confounding factor because their genotypes indicated less than 95% Holstein-Friesian background. GEBV for heat tolerance were calculated for the remaining animals using the prediction equation of Nguyen *et al.*^17^. Cows were ranked on GEBV for heat tolerance (accuracy for of the GEBV for milk yield decline (L) with increasing THI was 0.48), and the 24 most heat tolerant (mean GEBV 0.02, range 0.009 to 0.04) and 24 most heat susceptible (mean GEBV −0.02, range − 0.03 to −0.01) were purchased and relocated to the research centre.

The 48 cows were randomly assigned to 1 of 6 climate chambers in 8 cohorts each balanced for days in milk (DIM) and body weight (BW). At the beginning of the experiment for all of the cohorts, mean DIM was 67 for HT cows and 68 for HS cows and mean BW was 477 kg for heat tolerant cows and 488 kg for susceptible cows. The total length of the experiment for each cohort was 26 consecutive days, consisting of a 7 day baseline (BL) measurement period in ambient conditions; a 4-day heat challenge in controlled-climate chambers; and 14 day recovery period in ambient conditions. During all experimental periods cows had *ad libitum* access to a compressed cube with 74% DM lucerne hay (*Medicago sativa L.*), 25% DM crushed barley grain (*Hordeum vulgare L.*) and 1% minerals (calcium, phosphorus and magnesium). The cubed diet was formulated to meet the expected requirements of energy, protein and minerals (Supplementary Table 3)^29^. To ensure no bias was introduced into the experiment it was conducted as a blind experiment, meaning the HT and HS cows were not able to be identified by any of the researchers during the experiment. This was to ensure that the subjective measurements such as panting score were not influenced subconsciously by the knowledge that the animal was either HT or HS, and the decision to remove cows from the experiment based on the thresholds was also unbiased.

### Experimental facilities

The cows were introduced to the diet and outdoor feeding facility for 7 days prior to the experiment and were trained in, and desensitised to, the controlled-climate chambers for at least 4 weeks prior to the experiment. During the baseline and recovery periods the cows were housed in an outdoor, automatic feed-intake recording facility where they had 24 hour access to feed, water and bare paddocks for rest. The feed system identified the cows electronic ear tag and recorded the weight of the feed bin every second (Gallagher Animal Management Systems, Hamilton, New Zealand).

### Controlled-climate chambers

During the heat challenge period, the cows were housed individually in each climate chamber and were restrained by a head yoke which allowed them to stand up and lay down as well as turn their head around for grooming and sleeping. The cows had 2 padded mattresses to lay on (65 mm thick each), and free access to feed and water. Each chamber had windows (3780 × 1500 mm) in the walls between chambers which allowed for visual communication with the cows in the adjacent chambers.

The controlled-climate chambers were supplied and installed by No Pollution Industrial Systems (Edinburgh, Scotland). Each chamber is constructed from sandwich panels of polyurethane foam injected into a stainless steel outer shell and has a volume of 52 m^3^. Joins between panels and floor were sealed with Sikaflex PRO (Sika, Baar, Switzerland). The floor of each chamber is concrete which was sealed with 3 coats of Rhinofloor HBE 100% solids (Wagon Paints, Bayswater North, Vic, Australia) to prevent absorption of gas. Garnet was sprinkled between coats 1 and 2 to provide a non-slip surface. The air within each chamber is circulated independently at about 7.8 m^3^/min, being drawn from behind the animal and returned at the front of the animal stall. The circulated air is filtered and maintained at a programmed temperature and relative humidity. The condensed water from the dehumidifier is collected in an open container inside the chamber, allowing dissolved gases to vent back into the chamber. Air is exhausted from each chamber at 3.6 m^3^/min. Flow rate is measured using stainless-steel venturi tubes, individually calibrated for each chamber. The exhausted air is replaced with fresh air drawn through a large stainless-steel duct from outside the building. Temperature and relative humidity of the air entering and leaving the chambers as well as air circulation and exhaust rate were recorded by the system control software (Metasys; Johnson Controls, Milwaukee, WI, USA).

The conditions in the controlled-climate chambers were designed to remain above THI 72 and not exceed THI 84 to impose a moderate level of heat stress. The climatic conditions programmed into the control system were 25 °C and 60% RH (THI 74) between 6 PM and 6 AM, 30 °C and 50% RH (THI 80) between 6 AM and 12 noon, and 33 °C and 50% RH (THI 84) between 12 noon and 6 PM. The 12 hour light and 12 hour dark cycle was controlled manually. The THI was calculated using Equation (1)^30^;


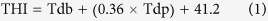


where: Tdb = hourly dry bulb temperature (°C); Tdp is dew point temperature (°C), Tdp = (237.3 × b)/(1.0 − b); b = [log(RH/100.0) + (17.27 × Tdb)/(237.3 + Tdb)]/17.27.

### Measurements and sampling

The physiological measurements recorded were respiration rate (breaths per minute, assessed by counting visible flank movements), panting score^31^, rectal temperature (212772 large animal digital thermometer; Shoof International Ltd, Cambridge, New Zealand), and skin temperature of the flank, neck and udder (AR300 + non-contact infrared thermometer, Smartsensor, Houston, USA). During the baseline and recovery periods, respiration rate, panting score, rectal and skin temperatures were recorded two times a day at 0600 and 1500 h, twice a week. During the heat challenge in the climate chambers, respiration rate and panting score were recorded three times daily at 0600, 1200 and 1500 h, and rectal and skin temperatures were recorded twice daily at 0600 and 1500 h on each day of the heat challenge. The area of skin where the measurement was taken was marked on each cow with spray paint so the measurement was taken from the same place each time. Intra-vaginal blank CIDR’s (Zoetis, Melbourne, Australia) were modified to house temperature recording buttons (DS1922L iButton; Thermodata, Warrnambool, Australia) and were inserted into the vagina of each cow for the duration of the experiment. Intra-vaginal temperature was recorded every 30 minutes during the baseline and recovery periods and every 15 minutes during the 4 day heat challenge period.

While in the controlled-climate chambers, cows were fed and orts were recorded twice daily. Samples of feed offered and refused were collected twice daily for dry matter and analysis of chemical composition. Daily samples were dried at 100 °C until constant weight for determination of dry matter. An additional subsample of the AM and PM daily feed offered was stored at −20 °C and bulked by week, then freeze dried and ground through a 1 mm screen for chemical composition analysis of DM, crude protein, neutral detergent fibre, acid detergent fibre, lignin, ash, starch and estimated metabolisable energy. Samples were analysed by near infrared spectroscopy at ACE Laboratory Services, Bendigo East, Victoria, 3550, Australia.

Cows were milked twice daily at (0600 and 1500 h), with yields recorded automatically at each milking during the baseline and recovery periods (MM25; DeLaval International, Tumba, Sweden). Cows were milked in the controlled-climate chambers and milk yields were recorded manually. Samples for milk composition analysis were taken from 6 milkings (3 × AM and 3 × PM) each week during baseline and recovery periods, and from each milking during the heat challenge. Samples were analysed for fat, protein, and lactose by a near-infrared milk analyser (model 2000, Bentley Instruments, Chaska, MN, USA). Somatic cells were counted by a Fossomatic SC300 cellcounter. Energy corrected milk (ECM) was calculated using equation (2)^32^;


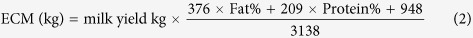


Water intake was recorded automatically in the controlled-climate chambers using flow meters in the water troughs. Bodyweights were recorded daily during the baseline and recovery periods using walk-over scales. Body condition score (BCS) was assessed using an 8 point scale^33^ once during the baseline and once during the recovery measurement periods.

### Statistical analysis

Statistical analyses were performed with ANOVA and general linear regression in GenStat (17^th^ edition; VSN International Ltd., Hemel Hempstead, UK). Residuals were examined graphically for normality of distribution and constant variance.

The following linear model was fitted for each variable measured and P values were determined (*y*, a vector of 48 measurements);


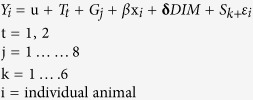


where **T**_**t**_ was a fixed effect of heat tolerance (n = 24) or heat susceptibility (n = 21) (as classified by the genomic prediction equation), **G**_**j**_ was a fixed effect of cohort 1 to 8, **x**_**i**_ was the baseline value for the Y variable measured during the covariate period for each animal, **DIM** was days in milk as at the beginning of the experiment, ***S***_***k***_ was where the animals were purchased from identified by geographic region within Victoria, Australia (n = 6), **ε**_**i**_ was the error term.

The standard error of the means (SEM) were determined using ANOVA fitted with heat tolerance/heat susceptibility as the treatment structure, and cow as the experimental unit with no blocking structure, and adjusted for the covariate. Trends identified at ^^^P < 0.10, and statistical difference identified at *P < 0.05, **P < 0.01, and ***P < 0.001.

An area under the curve (AUC) analysis was conducted with the vaginal temperature data to determine the °C.minutes that vaginal temperature was elevated above 39 °C during each day of the heat challenge, where for each cow the area under the vaginal temperature curve (minutes above 39 °C) was calculated, and was fitted into the liner model to determine if the AUC was significantly different between HT and HS cows.

## Additional Information

**How to cite this article**: Garner, J. B. *et al.* Genomic Selection Improves Heat Tolerance in Dairy Cattle. *Sci. Rep.* **6**, 34114; doi: 10.1038/srep34114 (2016).

## Supplementary Material

Supplementary Information
