# Corrigendum: Genomic Selection Improves Heat Tolerance in Dairy Cattle

**DOI:** 10.1038/srep39896

**Published:** 2017-01-19

**Authors:** J. B. Garner, M. L. Douglas, S. R. O Williams, W. J. Wales, L. C. Marett, T. T. T. Nguyen, C. M. Reich, B. J. Hayes

Scientific Reports 6: Article number: 34114; 10.1038/srep34114 published online: 09 29 2016; updated: 01 19 2017.

The original version of this Article omitted an affiliation for J. B. Garner. The correct affiliations are listed below:

Agriculture Victoria, Department of Economic Development, Jobs, Transport and Resources, 1301 Hazeldean Road, Ellinbank, Victoria 3821, Australia.

Faculty of Veterinary and Agricultural Sciences, The University of Melbourne, 142 University Street, Parkville VIC 3053, Australia.

This has now been corrected in the HTML and PDF versions of this Article.

