# Supplementary material for: Genomic Selection Improves Heat Tolerance in Dairy Cattle
Source: Sci Rep. 2016 Sep 29;6:34114. doi: 10.1038/srep34114 (PMC5040955; doi:10.1038/srep34114)
Supplement: Supplementary Information [file srep34114-s1.pdf]

Supplementary information - Nature Scientific Reports

Genomic selection improves heat tolerance in dairy cattle

J. B. Garner,[1]* M.L. Douglas,[1] S. R. O Williams,[1] W. J. Wales,[1] L. C. Marett [1], T. T. T. Nguyen,[2] C. M. Reich[2] and B. J. Hayes[2,3]

[1] Agriculture Research, Department of Economic Development, Jobs , Transport and Resources, 1301 Hazeldean Road, Ellinbank, Victoria 3821, Australia.

[2] BioSciences Research Division, Department of Economic Development, Jobs , Transport and Resources, AgriBio, 5 Ring Road, Bundoora, Victoria 3083, Australia.

[3] Queensland Alliance for Agriculture and Food Innovation, Centre for Animal Science, University of Queensland, Queensland, Australia

Supplementary Table 1. Daily means and standard error of the mean (SEM) of production and digestibility variables measured during the baseline (BL), heat challenge (HC) and recovery periods (RE) for the HT and HS cows. Trends identified at ^ P < 0.10, and statistical difference identified at * P < 0.05, ** P < 0.01, and *** P < 0.001

Period	BL (7 days)			HC day 1			HC day 2			HC day 3			HC day 4			RE (14 days)		
**Variable**	**HT**	**HS**	**SEM**	**HT**	**HS**	**SEM**	**HT**	**HS**	**SEM**	**HT**	**HS**	**SEM**	**HT**	**HS**	**SEM**	**HT**	**HS**	**SEM**
**DMI (kg)**	18.4	19.5	0.54	16.1	16.6**	0.89	15.1	15.5	0.6	15.9	14.8	0.7	14.4	13.2	0.64	19.7	19.8	0.41
**Milk yield (L)**	22	23.3	0.88	22.3	22.5	0.51	22.4	22.1	0.53	21.6	21.7	0.34	19.8	19	0.44	20.5	21.2	0.95
**ECM (kg)**	21.8	22.9	0.85	22.4	23.1	0.92	23.4	23.4	0.65	22.4	22.1	0.41	20.2	18.9^	0.42	20.4	21	0.9
**Fat %**	3.99	3.89	0.11	4.12	4.16	0.19	4.61	4.55	0.12	4.51	4.32	0.11	4.48	4.22	0.12	4.1	3.9	0.11
**Protein %**	3.22	3.23	0.05	3.22	3.21	0.02	3.16	3.11^	0.02	3.06	3.05	0.02	2.99	2.91	0.04	3.19	3.17	0.06
**Lactose %**	5.16	5.19	0.03	5.13	5.16^	0.02	5.12	5.17*	0.03	5.11	5.15**	0.02	5.18	5.14	0.07	5.1	5	0.07
**Fat, kg**	0.87	0.92	0.04	0.89	0.98	0.05	0.98	1.05*	0.03	0.94	0.96	0.02	0.86	0.82	0.02	0.82	0.84	0.03
**Protein, kg**	0.7	0.76	0.02	0.69	0.75***	0.01	0.68	0.72*	0.02	0.64	0.68***	0.01	0.58	0.56	0.02	0.66	0.69	0.03
**Lactose, kg**	1.13	1.23	0.04	1.11	1.21***	0.02	1.11	1.2**	0.03	1.08	1.16***	0.02	1	1.02	0.03	1.05	1.08	0.05
**Faeces (kg DM)**	na	na	na	6.3	6.6	0.25	5.9	6.1	0.25	5.9	6.3	0.23	5.6	5.7	0.24	na	na	na
**Urine (kg)**	na	na	na	19.5	20.5	0.91	16.6	20.3*	1.24	18	18.9	1.54	19	18.7	2.21	na	na	na
**DMD %**	na	na	na	63.9	62.2	1.46	61.1	60.2	1.51	61.9	60.1	2.19	60.5	58.8	1.5	na	na	na
**Water intake (L)**	na	na	na	79.5	84.4	3.97	74.6	85.5*	4.78	78.4	78.1	4.73	75.6	89.2*	6.13	na	na	na
**Bodyweight (kg)**	477	488	11.79	na	na	na	na	na	na	na	na	na	na	na	na	485	496*	12.04
**BCS**	4.62	4.59	0.04	na	na	na	na	na	na	na	na	na	na	na	na	4.63	4.61	0.03

Supplementary Table 2. Daily, morning and afternoon means, and standard error of the mean (SEM) of physiological variables measured during the BL, HC and RE periods for the HT and HS cows. Trends identified at ^ P < 0.10, and statistical difference identified at * P < 0.05, ** P < 0.01, and *** P < 0.001

Period	BL (7 days)			HC day 1			HC day 2			HC day 3			HC day 4			RE (14 days)		
Variable	HT	HS	SEM	HT	HS	SEM	HT	HS	SEM	HT	HS	SEM	HT	HS	SEM	HT	HS	SEM
**Daily**																		
**Vaginal, °C**	38.7	38.8	0.07	39.1	39.40**	0.07	39.3	39.80**	0.1	39.7	40.20**	0.1	39.9	40.30**	0.1	38.8	38.9	0.06
**Vaginal °C.m***	246	203	43.6	351	595*	80.6	525	1033**	127.6	1041	1672**	156.8	1333	1744**	164.3	225	270	38.2
**Rectal, °C**	38.3	38.2	0.06	39.2	39.5	0.1	39.6	40.10**	0.11	39.6	40.10**	0.11	39.9	40.21^	0.1	38.4	38.4	0.04
**Flank, °C**	23.1	21.3	0.62	37.2	36.5	0.46	38.5	37.00**	0.51	39.8	38.70**	0.37	40.1	38.40***	0.38	25.9	25.1	0.54
**Neck, °C**	28.6	28	0.57	40.8	40	0.46	42.2	40.80*	0.51	44	42.90*	0.41	44.2	42.50***	0.36	31.3	30.8	0.43
**Udder, °C**	35.4	36.5	0.47	42.7	41.80*	0.38	43.6	42.90*	0.41	44.6	44.6	0.31	44.8	44.1^	0.36	34.5	35.3	0.33
**Resp. rate^**	24	23	1.21	77.6	79.2	4.18	85.1	90.2^	3	87	95.00*	3.04	91.8	93	2.58	35.8	32.2	2.08
**Pant. score**	na	na	na	0.8	0.8	0.08	0.9	1.30*	0.08	1	1.1	0.11	1.1	1.4*	0.1	na	na	na
**Morning**																		
**Rectal, °C**	38.1	38.1	0.04	38.8	39.00*	0.09	38.9	39.40**	0.09	39.4	39.70*	0.1	39.6	39.8	0.1	38.2	38.1	0.03
**Flank, °C**	22.5	20.40*	0.7	37	36.5	0.6	38.6	37.00*	0.59	40.2	38.80*	0.46	41	38.90**	0.56	24.8	23.30***	0.59
**Neck, °C**	27.9	27.1	0.72	40.6	39.8	0.62	42.4	41.4	0.55	44.5	43.3	0.47	45	43***	0.43	29.9	29	0.48
**Udder, °C**	36	37.00**	0.4	43.1	41.90*	0.55	44.3	43.50**	0.5	45	45.2	0.4	45.4	44.6	0.58	35.9	36.60**	0.31
**Resp. rate^**	22.2	20.6	1.13	69.9	73.6	4.22	73	83.80**	3.17	73.8	80.5	3.82	81.6	77.8	3.58	22.6	21.9	0.74
**Pant. score**	na	na	na	0.7	0.9	0.11	0.8	1.1	0.09	0.5	0.8*	0.12	0.9	1	0.12	na	na	na
**Afternoon**																		
**Rectal, °C**	38.4	38.3	0.05	39.1	39.2	0.11	39.3	39.70**	0.12	39.8	40.40***	0.14	40.3	40.6^	0.12	38.7	38.60*	0.05
**Flank, °C**	23.8	23.2	0.88	37.6	36.9	0.53	38.4	36.90**	0.56	39.5	38.60^	0.47	39.1	37.80**	0.45	27.1	25.80**	0.66
**Neck, °C**	29.5	30.2	0.72	41.1	40.3	0.47	42.1	40.70**	0.53	43.6	42.40*	0.44	43.4	41.90**	0.43	31.8	31	0.45
**Udder,°C**	34.3	35.40**	0.73	42.4	41.7	0.39	42.9	42.3	0.47	44.2	44.1	0.38	44.3	43.5	0.32	33.3	34^	0.37
**Resp. rate^**	25.5	24.4	1.33	94.5	96.5	5.12	95.2	100.8	3.7	100.6	108.1	3.87	103.3	107.8	2.75	49.1	42.50***	3.62
**Pant. score**	na	na	na	0.9	1.2	0.11	1.2	1.3	0.11	1.3	1.6	0.16	1.6	1.8	0.15	na	na	na

*Area under the curve for vaginal temperature °C.minutes above 39°C. ^ respiration rate measured as breaths per minute.

Supplementary Table 3. Dry matter (DM), crude protein (CP), neutral detergent fibre (NDF), acid detergent fibre (ADF), lignin, ash, starch and estimated metabolisable energy (ME) (MJ/kg) in the lucerne and barley cubes. Data are means for samples collected each day during the experiment. Units are % DM unless otherwise stated.

Item	Lucerne cubes w/ barley	
	Mean	s.d.
DM (%)	94.7	0.16
CP	18.1	0.38
NDF	39.2	0.9
ADF	30.7	0.89
Lignin	6.7	0.2
Ash	7.7	0.41
Starch	6.8	0.24
ME (MJ/kg)	9.9	0.1

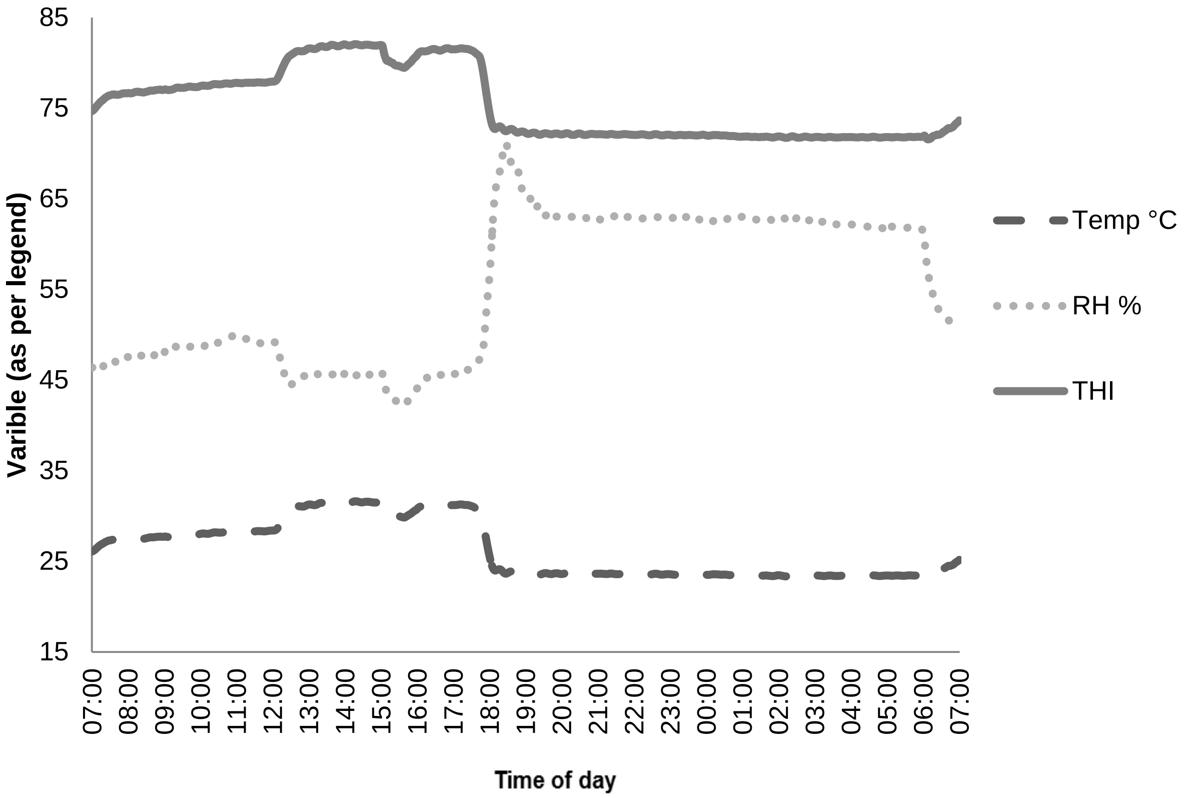

Supplementary Figure 1. Mean of the six controlled-climate chambers for each 24-hour period during the 4-day heat challenge (8 heat challenge periods for the 8 groups of cows) for THI (SEM = 0.05), temperature (SEM = 0.03) and relative humidity (SEM = 0.13) during 24 hours.
